# The effect of physical activity on growth factor concentrations in platelet-rich fibrin: a cross-sectional study

**DOI:** 10.1590/1678-7765-2026-0128

**Published:** 2026-06-18

**Authors:** Elif Yucel, Meltem Karsiyaka Hendek, Hakan Yapici, Mehmet Gulu, Osman Caglayan, Ebru Olgun

**Affiliations:** 1 Kirikkale University Faculty of Dentistry Department of Periodontology Kirikkale Turkey Kirikkale University, Faculty of Dentistry, Department of Periodontology, Kirikkale, Turkey; 2 Kirikkale University Faculty of Sport Sciences Department of Recreation Kirikkale Turkey Kirikkale University, Faculty of Sport Sciences, Department of Recreation, Kirikkale, Turkey; 3 Kirikkale University Faculty of Sport Sciences Department of Sport Management Kirikkale Turkey Kirikkale University, Faculty of Sport Sciences, Department of Sport Management, Kirikkale, Turkey; 4 Kirikkale University Faculty of Medicine Department of Medical Biochemistry Kirikkale Turkey Kirikkale University, Faculty of Medicine, Department of Medical Biochemistry, Kirikkale, Turkey

**Keywords:** International physical activity questionnaire, Growth factors, Physical activity, PRF, Platelets

## Abstract

**Objective:**

This study investigated the impact of physical activity levels on growth factor concentrations within leukocyte- and platelet-rich fibrin (L-PRF).

**Methodology:**

In total, 120 participants were categorized into sedentary (n=60) and physically active (n=60) groups via the International Physical Activity Questionnaire. Venous blood was collected to analyze platelet count, mean platelet volume, white blood cell count, red blood cell count, plateletcrit, platelet distribution width, and platelet-large cell ratio, and to prepare the L-PRF. Concentrations of vascular endothelial growth factor (VEGF), transforming growth factor-beta (TGF-β), and platelet-derived growth factor (PDGF) were determined using ELISA.

**Results:**

Metabolic equivalent of task (MET) values were significantly higher in the active group (p<0.001). Conversely, VEGF, TGF-β, and PDGF levels were significantly higher in the sedentary group (p<0.001). Hemoglobin and hematocrit values were also significantly higher in active individuals (p=0.025 and p=0.040, respectively). A significant moderate negative correlation was found between MET values and concentrations of VEGF, TGF-β, and PDGF (r=−0.33, r=−0.33, and r=−0.31, respectively; p<0.01). Multivariable linear regression analyses, adjusted for sex, periodontal parameters, and hematological variables, confirmed that Total MET constituted an independent negative predictor of all growth factors (all p<0.001), whereas periodontal indices showed no significant independent associations. Sex-stratified analyses and interaction models showed that the association between physical activity and growth factor levels was significantly sex dependent (MET × sex interaction: all p<0.05), with stronger effects in women.

**Conclusions:**

Physical activity seems to be associated with variations in growth factor levels in L-PRF, suggesting that individual physiological status may influence the biological composition of autologous platelet concentrates.

## Graphical abstract

**Figure:**
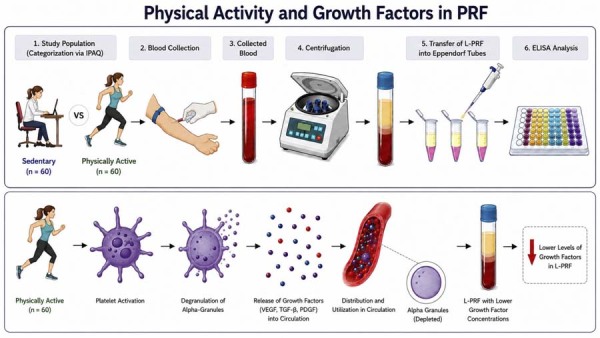

## INTRODUCTION

Physical activity is defined as any bodily movement produced by skeletal muscles that requires energy expenditure.^1^ Physical activity can be performed during occupational tasks, household activities, transportation, leisure time pursuits, or structured exercise and sports activities. Regular physical activity plays a significant role in the prevention and management of various diseases and conditions, including cardiovascular diseases, stroke, diabetes, breast and colorectal cancers, and osteoporosis.^1,2^ Physical activity also provides important benefits for mental and cognitive health, including delaying the onset of dementia.^3,4^ It promotes the attainment of a healthy body weight and supports the maintenance of overall well being.^5^ Sedentary behavior is defined as waking periods spent sitting, reclining, or lying down with minimal energy expenditure.^6^ High levels of sedentary behavior are associated with increased mortality related to various chronic diseases.^7,8^ The International Physical Activity Questionnaire (IPAQ), an internationally valid survey, was designed to assess individuals’ physical activity levels. This questionnaire was developed to meet the needs of health policy making institutions such as the World Health Organization and to facilitate the monitoring of physical activity levels within populations. Its short (seven items) and long (27 items) forms were developed to assess physical activity in individuals aged 18–65 years.^9^ Using this questionnaire, which examines individuals’ walking, moderate or vigorous intensity physical activity, and sitting time during the preceding seven days, their physical activity level is calculated in metabolic equivalents (MET minutes/week). These values are used to classify individuals as having low, moderate, or high levels of physical activity.^10^ Platelet-rich fibrin (PRF) is an autologous second generation platelet concentrate obtained from patients’ own blood, characterized by its low risk of immune reaction and the absence of additives.^11^ PRF accelerates the healing of periodontal tissues by supporting key biological processes such as cell proliferation, angiogenesis, matrix synthesis, and bone regeneration.^12^ Exercise has been shown to influence the cellular composition of blood and platelet-rich plasma.^13^ Physical activity may alter the quantity of growth factors by influencing the number and function of circulating platelets.^14^ Additionally, physical activity affects the immune system and inflammatory response by increasing circulating leukocyte counts.^15^ The positive effects of physical activity on cellular metabolism may be reflected in the biological quality of PRF.^16^ In line with this background, this study aimed to investigate the effect of physical activity on the growth factors within leukocyte and platelet-rich fibrin (L-PRF). The hypothesis of the study was formulated as follows: “Physical activity reduces the levels of growth factors within L-PRF.”

## METHODOLOGY

In this cross-sectional observational study, participants were recruited from systemically healthy individuals seeking periodontal treatment at the Department of Periodontology, Kirikkale University, Faculty of Dentistry, Kirikkale, Turkey, from January 15, 2025, to June 15, 2025. They were screened for eligibility according to the inclusion and exclusion criteria in this study. The study protocol was approved by the Institutional Non-Interventional Research Ethics Committee at Kirikkale University (18.12.2024, Decision No: 2024.12.14).

### Patient selection

All individuals in this study were informed of its purpose and methodology. Written consent was obtained using an informed voluntary consent form approved by the ethics committee. Systemically healthy individuals with good cooperation and the ability to comply with the study procedures and those who were mentally competent were included in this study. In contrast, individuals who smoked and/or consumed alcohol; those with hematological disorders that could affect platelet function such as thrombocytopenia or thrombocytosis; those using anticoagulant, coagulant, or steroid medications; and pregnant or breastfeeding individuals were excluded from the study. A total of 120 participants were evaluated using the IPAQ and then divided into two groups: 60 individuals classified as highly physically active (test group) and 60 individuals classified as sedentary (control group). Physical activity levels were classified according to the standard IPAQ scoring protocol (based on MET minutes/week thresholds).

### Clinical evaluation

All clinical measurements were performed by a single calibrated examiner to minimize potential bias. The periodontal status of all participants was assessed and recorded using probing depth (PD), clinical attachment level (CAL), plaque index (PI) (Silness & Löe, 1964)^17^, gingival index (GI) (Löe & Silness, 1963)^18^, and bleeding on probing (BOP). Periodontal parameters were recorded to control for potential confounding effects of inflammatory status on growth factor measurements.

### Collection of blood samples and preparation of leukocyte and platelet- rich fibrin

A total of 20 mL of venous blood (two additive free glass tubes containing 10 mL each) was drawn from the antecubital vein of each participant. Participants were instructed to avoid vigorous physical activity for at least 48 hours prior to blood collection. The first tube was used for complete blood count analysis to determine hematological parameters. Platelet count (PLT), mean platelet volume (MPV), white blood cell count (WBC), red blood cell count, plateletcrit, platelet distribution width (PDW), and platelet large cell ratio were measured in this analysis. The second tube was used to obtain leukocyte and platelet rich fibrin (L-PRF) to determine growth factor levels via enzyme-linked immunosorbent assay. After blood collection, the tubes were centrifuged at 400 g, 2700 rpm for 12 minutes (Hettich EBA 20 Centrifuge, Germany). Following centrifugation, the L-PRF clots were removed from the tubes using sterile forceps. The erythrocyte layer at the lower portion of the L-PRF clot was removed using sterile scissors, and the remaining L-PRF clots were transferred into new red topped glass tubes. These tubes were then centrifuged at 1500 g, 4000 rpm for 10 minutes (NF 1200R, Turkey), to obtain the supernatant. This secondary centrifugation step was performed to remove cellular components (platelets, leukocytes, and residual fibrin fragments) from the PRF matrix and to obtain a cell free, matrix-derived supernatant suitable for quantitative enzyme-linked immunosorbent assay analysis.^19,20^ The supernatants from the L-PRF samples were transferred into Eppendorf tubes using Pasteur pipettes and stored at −80 °C up to the day of analysis. The concentrations of growth factors within the L-PRF samples were determined using Human TGF-β (YL Biont, Shanghai YL Biotech Co., Ltd., Cat. No: YLA0886HU, China), Human PDGF (YL Biont, Shanghai YL Biotech Co., Ltd., Cat. No: YLA1163HU, China), and Human VEGF (YL Biont, Shanghai YL Biotech Co., Ltd., Cat. No: YLA1352HU, China) ELISA kits based on a biotin double antibody sandwich enzyme immunoassay. All laboratory procedures and measurements were performed using identical protocols for both groups.

Standard calibration curves were prepared for each growth factor using serial dilutions of recombinant standards with known concentrations. Optical density was measured at 450 nm using a microplate reader. Linear regression analysis was used to determine the relationship between absorbance and sample concentration. The regression equations were as follows: $\text{ VEGF: }y=441.94x+8.8117\left(R^{2}=0.9981\right)$ , $\text{ TGF- }\beta :y=1923.4x+44.953\left(R^{2}=0.9986\right)$ , $\text{ PDGF: }y=300.47x+1.3272\left(R^{2}=0.9994\right)$ , in which x represents absorbance and y, concentration (ng/L).

Sample concentrations within the standard curve range were calculated by interpolation using the equations above. For samples with absorbance values exceeding the highest standard concentration, concentrations were calculated by extrapolation using the same regression equations.

### Statistical analysis

The sample size required for the study was determined on G-Power (G-Power Version 3.0.10, Franz Faul, Universität Kiel, Germany). It was calculated that a minimum of 118 participants would be required for an effect size of f = 0.5, a Type I error probability of α = 0.05, and a statistical power of 0.85. All data obtained within the scope of this study were analyzed on IBM SPSS Statistics, 25.0. The Shapiro–Wilk normality test was performed to determine whether the data followed a normal distribution. Analyses showed that all variables had a normal distribution. Therefore, independent samples t tests were used for comparisons between the groups. Continuous variables are shown as mean ± standard deviation (min–max), whereas categorical variables, as n (%). Gender distribution was evaluated using the chi-squared test. A significance level of p<0.05 was adopted to evaluate differences between the groups. The magnitude of the observed differences was quantified using Cohen’s d effect size, interpreted according to the conventional benchmarks proposed by Cohen (1988): values of 0.2, 0.5, and 0.8 were considered indicative of small, medium, and large effects, respectively. Additionally, Pearson’s correlation analysis was performed to determine the relationships between the variables. The relationships between growth factors, MET scores, hematological parameters, and clinical measurements were evaluated ın the correlation analysis (significance levels of p<0.05 and p<0.01 were considered as meaningful associations). Furthermore, multivariable linear regression analyses were conducted to evaluate the independent effects of relevant variables. Model diagnostics — including the Durbin–Watson statistic, residual distribution, and multicollinearity (variance inflation factor) — were assessed to ensure the validity of the regression models.

## RESULTS

### Demographic characteristics and MET values

The mean age of the test group was 23.07±1.98 years, whereas the mean age of the control group, 22.72±2.02 years. Participants’ mean age did not differ significantly between the test and control groups. An examination of the gender distribution showed that the control group consisted of 33 women (47.8%) and 27 men (52.9%), whereas the test group consisted of 36 women (52.2%) and 24 men (47.1%). This study found no statistically significant difference between the groups regarding gender distribution (p=0.712) (Table 1). The mean MET value totaled 4501±1355 in the test group and 323±68 in the control group. Total MET values significantly differed between the test and control groups (p<0.05). MET values significantly differed between physically active and sedentary women and between physically active and sedentary men (p<0.05) (Table 1).

**Table 1:** Comparison of demographic characteristics and MET values between the physically active and sedentary groups

	Physically Active Group	Sedentary Group	p	t
**Age (Mean ± sd)**	23.07 ± 1.98	22.72 ± 2.02	0.340	
**Gender (n, %)**			0.712	
**Female**	36 (52.2)	33 (47.8)		
**Male**	24 (47.1)	27 (52.9)		
**MET (mean ±sd)**				
**Total**	4501±1355	323±68	< 0.001*	-23.840
**Female**	4126±1159	325±59	< 0.001*	-19.655
**Male**	4960±1456	320±80	< 0.001*	-15.573

Sd: Standard Deviation, MET: Metabolic Equivalent of Task

*, Statistically significant difference between groups (p<0.05)

No missing data were observed for any variable in the analyses.

### Periodontal clinical parameters

No statistically significant differences were found between the physically active and sedentary groups regarding PD, GI, or CAL (p>0.05). These parameters showed small effect sizes (Cohen’s d = 0.042, 0, and 0, respectively). However, BOP and PI values were significantly higher in the physically active group than in the sedentary group (p=0.013 and p=0.006, respectively), with a small effect size for BOP (Cohen’s d = 0.47) and a medium effect size for PI (Cohen’s d = 0.6) (Table 2).

**Table 2:** Clinical Periodontal Parameters in the physically active and sedentary groups

Clinical Parameters	Groups	Mean ± sd	t	p	Cohen’s d	Descriptor
**PD**	Sedentary	1.74± 0.3	-0.114	0.909	0.042	Small
	Physically Active	1.76± 0.6				
**CAL**	Sedentary	1.74± 0.3	0.298	0.766	0	Small
	Physically Active	1.76± 0.6				
**PI**	Sedentary	1± 0.5	-2.778	0.006*	0.6	Medium
	Physically Active	1.4± 0.8				
**GI**	Sedentary	1± 0.3	0.298	0.766	0	Small
	Physically Active	1± 0.4				
**BOP**	Sedentary	8.5± 9	-2.533	0.013*	0.47	Small
	Physically Active	15.5± 19				

Sd: Standard Deviation, PD: Probing Depth, CAL: Clinical Attachment Level, PI: Plaque Index, GI: Gingival Index, BOP: Bleeding on Probing

*, Statistically significant difference between groups (p<0.05)

In female participants, the evaluation of clinical periodontal parameters revealed that PD and CAL values were statistically significantly higher in physically active women than in sedentary ones (p=0.03), with a large effect size for both variables (Cohen’s d = 0.8). GI was also found to be statistically significantly higher in physically active women than in sedentary ones (p=0.05), with a small effect size for this difference (Cohen’s d = 0.48). On the other hand, no statistically significant differences were observed between the groups regarding BOP and PI values. These findings were supported by a small effect size. All clinical periodontal parameter values were higher in physically active male participants than in sedentary men. These differences were statistically significant (p<0.05). Notably, the Cohen’s d value for GI and PI measurements totaled 1.5, indicating a large effect size. Moreover, the effect size for BOP was large (Cohen’s d = 0.86), as those for PD and CAL were also large (Cohen’s d = 0.6).

### Growth factor concentrations

**Figure 1:**
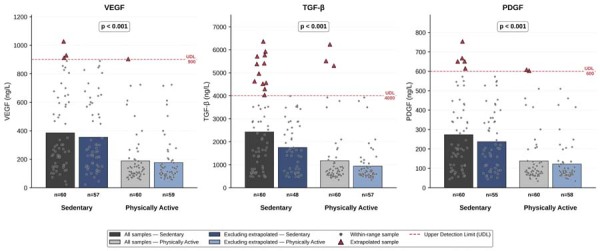
Comparison of growth factor concentrations in sedentary and physically active participants with and without extrapolated samples. Bars represent mean concentrations with individual datapoints for each participant. Gray circles indicate samples within the assay range, whereas red triangles represent samples above the upper detection limit (UDL). Dashed red lines indicate assay-specific UDLs. The numbers of extrapolated samples refer to VEGF: n = 4 (3 sedentary, 1 physically active), TGF-β: n = 15 (12 sedentary, 3 physically active), and PDGF: n = 7 (5 sedentary, 2 physically active). The groups were compared using independent-samples t-tests.

The VEGF, TGF-β, and PDGF levels of the sedentary group were found to be statistically significantly higher than those of the physically active group (Figure 1). The Cohen’s d effect size values exceeded 0.80 for all three variables, indicating a large effect size (Cohen’s d = 0.85, 0.83, and 0.82, respectively) (Table 3). Growth factor concentrations were statistically significantly higher in sedentary women than in physically active ones; these differences were supported by large effect sizes (Cohen’s d = 1.3–1.4). Growth factor concentrations were also statistically significantly higher in sedentary men than in physically active men. However, effect sizes were smaller than those in women, ranging from small to medium (Cohen’s d = 0.2–0.4).

**Table 3:** Comparison of growth factor levels between the physically active and sedentary groups

Growth Factors	Groups	Mean ± sd	t	p	Cohen’s d	Descriptor
**VEGF**	Sedentary	385± 272	4.645	< 0.001*	0.85	Large
	Physically Active	188± 182				
**TGF-β**	Sedentary	2427± 1688	4.567	< 0.001*	0.83	Large
	Physically Active	1176± 1284				
**PDGF**	Sedentary	273± 189	4.488	< 0.001*	0.82	Large
	Physically Active	137± 135				

Sd: Standard Deviation, VEGF: Vascular Endothelial Growth Factor, TGF-β:

Transforming Growth Factor-Beta, PDGF: Platelet Derived Growth Factor

*, Statistically significant difference between groups(p<0.05)

### Hemogram parameters

PLT, MPV, WBC, red blood cell count, plateletcrit, PDW, and platelet large cell ratio showed no statistically significant differences between the groups, and the effect sizes for all these parameters were small (Cohen’s d < 0.2). However, the hemoglobin (HGB) and hematocrit (HCT) values were significantly higher in physically active individuals than in sedentary ones (p=0.025 and p=0.040, respectively), with small effect sizes for both parameters (Cohen's d = 0.44 and 0.21, respectively) (Table 4).

**Table 4:** Hemogram parameter comparison between the physically active and sedentary groups

Hemogram Groups		Mean ± sd	t	p	Cohen’s d	Descriptor
PLT	Sedentary	270± 54	1.058	0.292	0.19	Small
	Physically Active	260± 52				
**MPV**	Sedentary	10.3± 1.0	-0.189	0.850	0.11	Small
	Physically Active	10.4± 0.9				
**WBC**	Sedentary	7.3± 1.7	-0.133	0.895	0.06	Small
	Physically Active	7.4± 1.4				
**RBC**	Sedentary	4.8± 0.5	-1.002	0.319	0.22	Small
	Physically Active	4.9± 0.4				
**HGB**	Sedentary	13.9± 1.7	-2.275	0.025*	0.44	Small
	Physically Active	14.6± 1.5				
**HCT**	Sedentary	42.2± 3.9	-2.082	0.040*	0.21	Small
	Physically Active	43± 3.9				
**PCT**	Sedentary	0.27± 0.06	-0.270	0.778	0.049	Small
	Physically Active	0.27± 0.05				
**PDW**	Sedentary	16.18± 0.4	-0.773	0.441	0.05	Small
	Physically Active	16.2± 0.3				
**PLCR**	Sedentary	29± 7.1	-0.146	0.884	0.028	Small
	Physically Active	29.2± 7				

Sd: Standard Deviation, PLT: Platelet Count, MPV: Mean Platelet Volume, WBC: White Blood Cell Count, RBC: Red Blood Cell Count, HGB: Hemoglobin, HCT: Hematocrit, PCT: Plateletcrit, PDW: Platelet Distribution Width, PLCR: Platelet Large Cell Ratio

*, Statistically significant difference between groups (p<0.05)

### Correlation analyses

**Figure 2:**
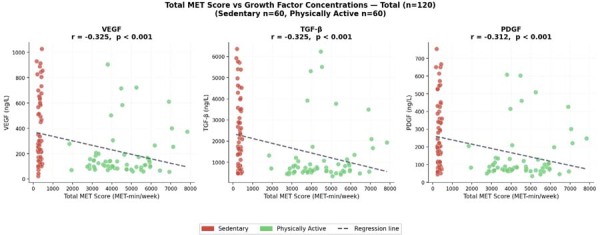
Association between total MET score and growth factor concentrations. Scatter plots illustrating the association between total MET scores and VEGF, TGF-β, and PDGF concentrations in all participants (n = 120). Sedentary participants are shown in red and physically active participants in green. Dashed lines represent the fitted linear regression lines. Pearson’s correlation coefficients and corresponding p-values are shown for each analysis.

Significant, moderate, and negative correlations were observed between MET scores and VEGF, TGF-β, and PDGF levels (r = −0.33, r = −0.33, r = −0.31, p < 0.01). (Figure 2). Weak and statistically non-significant negative correlations were observed between WBC and VEGF (r = –0.085), TGF-β (r = –0.057), and PDGF (r = –0.013). A significant but low level positive correlation was identified between PLT and PDGF (r = 0.192, p < 0.05). Although the correlations between PLT and VEGF (r = 0.155) and between PLT and TGF-β (r = 0.159) were positive, they were statistically insignificant. Weak negative correlations were identified between MPV and VEGF (r = –0.068), TGF-β (r = –0.117), and PDGF (r = –0.106). However, these associations were statistically insignificant (Table 5).

**Table 5:** Correlation analysis between MET values and growth factor concentration

MET	Growth Factor	Pearson r	p
**Total**	VEGF	-0.325	< 0.001*
	TGF-β	-0.325	< 0.001*
	PDGF	-0.312	< 0.001*
**Female**	VEGF	-0.498	< 0.001*
	TGF-β	-0.518	< 0.001*
	PDGF	-0.510	< 0.001*
**Male**	VEGF	-0.181	0.205
	TGF-β	-0.148	0.300
	PDGF	-0.139	0.330

MET: Metabolic Equivalent of Task, VEGF: Vascular Endothelial Growth Factor, TGF-β: Transforming Growth Factor-Beta, PDGF: Platelet Derived Growth Factor, *, Statistically significant difference between groups (p<0.05)

### Multivariable regression and sex interaction analyses

Multivariable linear regression analyses were performed to evaluate the independent association between physical activity and growth factor levels while adjusting for sex, PI, BOP, PLT, and MPV.

All models were statistically significant (VEGF: p = 0.005; TGF-β: p = 0.005; PDGF: p = 0.003). Total MET showed a significant negative association with all growth factors (all p<0.001). In contrast, PI, BOP, sex, and MPV showed no significant association with growth factor levels in any model (all p>0.05), whereas PLT only showed a significant positive association for PDGF (p=0.049).

Additional models including a MET x sex interaction term were constructed to assess potential sex-specific effects. All interaction models were statistically significant (VEGF: model p = 0.002; TGF-β: model p = 0.002; PDGF: model p<0.001). The MET x sex interaction term showed a significant positive association for all growth factors (VEGF: p = 0.041; TGF-β: p = 0.028; PDGF: p = 0.026), whereas Total MET remained a significant negative predictor (VEGF: p = 0.002; TGF-β: p = 0.001; PDGF: p = 0.001).

In these interaction models, PI, BOP, sex, and MPV were statistically insignificant predictors (all p>0.05), whereas PLT only remained significant for PDGF (p = 0.036).

## DISCUSSION

PRF plays a crucial role in wound healing and tissue regeneration in regenerative applications within dentistry and medicine owing to its content of leukocytes, platelets, and the growth factors released from these platelets. The quantity of growth factors within PRF and the structural characteristics of its membranes may vary depending on individual factors (such as age, sex, systemic conditions, smoking habits, and lifestyle) and differences in preparation protocols.^21,22^Regular physical activity can significantly affect hematological parameters, platelet function, circulating cytokines, and growth factor levels. Platelet activation increases during exercise. It has been shown that TGF-β1 and VEGF levels within platelet-rich plasma (PRP) are lower in professional athletes than in sedentary individuals.^23^ Similarly, another study reported that the levels of VEGF, TGF-β, and PDGF within PRP were lower in female athletes than in sedentary women.^24^ Another study has shown that exercise decreases the levels of growth factors within PRP.^25^ Czarkowska-Paczek, Bartlomiejczyk and Przybylski found that serum levels of PDGF, TGF-β, and VEGF increased following intense physical activity.^26^

During the physical stress induced by exercise, platelets are exposed to adrenergic stimuli, leading to their activation and the following release of stored growth factors from alpha granules.^27,28^ Considered together with studies reporting lower growth factor concentrations in platelet concentrates from professional athletes when compared to sedentary individuals and studies showing increased serum growth factor levels following physical activity, this pattern may suggest a redistribution of growth factors from platelets to the circulation.

In this context, it is plausible that repeated platelet activation associated with regular physical activity may influence the balance between platelet-stored and circulating growth factors. The lower growth factor concentrations in physically active individuals in this study may be associated with this mechanism.

However, as serum growth factor levels were not measured in this study, this interpretation cannot be directly verified, and should be considered as a hypothesis. Further studies incorporating simultaneous assessments and comparisons of serum- and PRF-derived growth factor levels are required to explain this relationship.

A subset of samples, predominantly from the sedentary group, exceeded the upper detection limits of the standard curves, and concentration values for these samples were estimated by extrapolation. This should be considered a methodological limitation. However, it probably introduced a conservative bias, suggesting that the true differences between the physically active and sedentary groups may be even greater than those reported here. A study investigating the immune responses of physically active and sedentary girls reported no significant differences in WBC values between the groups.^29^ Moreover, another study with physically active and sedentary individuals compared the WBC, HGB, and HCT values of both groups before and after exercise. In this study, no significant difference was found in WBC values between the two groups, whereas HGB and HCT levels were higher in physically active individuals.^30^ Substantial evidence indicates that physical activity, exercise, and various forms of sport can exert diverse effects on platelet counts and the parameters that reflect platelet characteristics. While some studies have shown that MPV values decrease following exercise,^31,32^ other studies have reported an increase in MPV values after exercise.^33^However, other studies have indicated that platelet counts remain the same following exercise.^34-36^ Kırbaş and colleagues, comparing athletic individuals and sedentary university students, found no significant difference in PDW values, a parameter reflecting platelet characteristics.^37^ Similarly, in this study, no significant differences were observed between physically active and sedentary individuals regarding platelet counts or the parameters reflecting platelet characteristics. Additionally, HGB and HCT values were higher in physically active individuals. In the study by Demircioğlu and colleagues, which compared body composition and erythrocyte mass between individuals who engaged in regular exercise and sedentary individuals, HGB and HCT values were likewise found to be higher in the physically active group.^38^ In our study, the higher HGB and HCT values in physically active individuals (when compared to sedentary individuals) may be explained by the adaptations physical activity induces in the cardiorespiratory system. Regular exercise stimulates erythropoietin secretion, increasing erythrocyte production and enhancing total hemoglobin mass, which improves oxygen-carrying capacity.^39^ An enhanced oxygen-carrying capacity may positively contribute to the healing and regeneration processes of periodontal tissues. In particular, the effectiveness of platelet-derived growth factors in PRF applications may be further enhanced when accompanied by increased tissue oxygenation capacity. Therefore, the findings of our study suggest that physical activity may have indirect beneficial effects on PRF applications in dentistry. An additional finding of this study is the potential influence of sex on the relationship between physical activity and PRF-derived growth factor levels. Its sex-stratified analyses showed that statistically significant inverse associations between TOTAL MET and all three growth factors were observed in women, whereas no significant associations were identified in men. Consistently, its interaction models indicated that the MET × sex interaction term was statistically significant for all three growth factors (VEGF: p = 0.041; TGF-β: p = 0.028; PDGF: p = 0.026).

These findings suggest that the association between physical activity and growth factor levels may be sex dependent rather than universal. Notably, no significant sex differences were observed within the sedentary group, whereas the women in the physically active group showed significantly lower growth factor concentrations than men. This pattern may indicate that sex-related biological differences become more apparent under exercise adaptation.

The underlying mechanisms are yet to be fully understood. However, sex-related differences in platelet biology may provide a plausible explanation. Previous studies have shown that female platelets may exhibit higher baseline reactivity than male platelets, potentially influenced by sex hormones, particularly estrogen.^40^ In contrast, Xiong, et al.^41^ (2018) reported that men may have higher baseline levels of growth factors such as TGF-β1, PDGF, and VEGF in platelet-rich preparations.

Taken together, these findings raise the possibility that repeated exercise-induced platelet activation may differentially influence growth factor dynamics in women and men. In women, heightened platelet responsiveness potentially modulated by hormonal factors may contribute to a more pronounced alteration in PRF-derived growth factor content following regular physical activity.

However, it is important to emphasize that these mechanistic interpretations remain speculative as platelet activation markers and circulating growth factor levels were not assessed in this study. Further studies incorporating sex-specific platelet activation markers and growth factor profiling are required to explain the biological pathways underlying these observations.

In this study, when the periodontal clinical parameters of physically active and sedentary individuals were compared, no statistically significant differences were found between groups regarding PD, CAL, or GI. We believe that the absence of significant differences in PD and CAL may be attributed to the young and relatively homogeneous age profile of the individuals in our study. However, in our study, significant differences were observed between physically active and sedentary individuals regarding BOP and PI. The higher plaque accumulation in physically active individuals and the corresponding increase in BOP that reflects gingival inflammation suggest that oral hygiene habits may be insufficient in physically active individuals. Moreover, products consumed during or prior to intensive training sessions may increase intraoral acidity, predisposing individuals to gingival inflammation.^42^ Also, mouth breathing and oral dryness during exercise may reduce the protective functions of saliva and facilitate plaque accumulation, contributing to gingival inflammation. A limitation of this study is that the release kinetics of growth factors from the fibrin matrix were not evaluated. Future studies investigating the effects of physical activity on the time-dependent release of growth factors from PRF would provide valuable insights and represent an important avenue for further research.

The reliance on a self-reported questionnaire to assess physical activity levels may also be considered a methodological limitation of this study. Although the validated IPAQ was used to standardize data collection, self-reported measures are inherently susceptible to recall and social desirability biases, which may contribute to the misclassification of activity levels. Future studies incorporating objective physical activity monitoring tools such as accelerometers or wearable devices alongside the IPAQ would substantially strengthen the validity of physical activity classification.

Furthermore, the physical dimensions, weight and volume of PRF clots were not recorded in this study. Consequently, the current findings reflect growth factor concentrations in the analyzed supernatant fraction rather than the total growth factor content per PRF clot. Future investigations incorporating weight and volumetric measurements are warranted to enable a more comprehensive and accurate evaluation of PRF composition.

Finally, dietary habits were not assessed in this study. They may represent a potential source of residual confounding. Future studies with validated dietary assessment instruments are needed to better elucidate the independent and combined effects of diet and physical activity on PRF-derived growth factor levels. Moreover, controlled experimental models (such as animal studies) utilizing standardized dietary and exercise protocols may yield further mechanistic insights into these relationships.

## CONCLUSION

According to the findings of this cross-sectional study, physical activity was associated with lower growth factor levels in L-PRF. However, given the observational design of this study, this relationship should be interpreted with caution, and does not imply a causal effect. In clinical applications involving PRF in regenerative therapies, physical activity may represent a potential influencing factor. However, further studies are required to determine their impact on fibrin structure, growth factor release kinetics, and clinical healing outcomes. Therefore, the clinical implications of these findings should be interpreted with caution.

Future studies should investigate the effects of physical activity on growth factor levels across different PRF types and preparation protocols. Furthermore, since PRF is frequently utilized in older clinical populations, future research must validate these findings in diverse age groups, specifically focusing on healthy middle-aged and older adults and patients with periodontal disease.
